# Cutaneous Intravascular Hematolymphoid Entities: A Review

**DOI:** 10.3390/diagnostics14070679

**Published:** 2024-03-23

**Authors:** Emily Hatheway Marshall, Bethany Brumbaugh, Allison Holt, Steven T. Chen, Mai P. Hoang

**Affiliations:** 1 Department of Pathology, Massachusetts General Hospital, Boston, MA 02114, USA; ehathewaymarshall@mgb.org; 2 Department of Dermatology, Massachusetts General Hospital, Boston, MA 02114, USA; bethany_brumbaugh@hms.harvard.edu (B.B.); allison.holt@umassmed.edu (A.H.); stchen@mgb.org (S.T.C.); 3 Harvard Medical School, Boston, MA 02115, USA; 4 University of Massachusetts Chan Medical School, Worcester, MA 01655, USA

**Keywords:** intravascular, intralymphatic, lymphoma, B-cell, T/NK-cell, histiocytosis, CD30

## Abstract

Intravascular lymphomas are rare disease conditions that exhibit neoplastic lymphoid cells that are confined mainly to the lumens of small capillaries and medium-sized vessels. The majority of the intravascular lymphomas are of B-cell origin, but they can include NK/T-cell and CD30+ immunophenotypes. In the histologic differential diagnosis are benign proliferations such as intralymphatic histiocytosis and intravascular atypical CD30+ T-cell proliferation. In this review, we discuss the clinical, histopathologic, and molecular findings of intravascular B-cell lymphoma, intravascular NK/T-cell lymphoma, intralymphatic histiocytosis, and benign atypical intravascular CD30+ T-cell proliferation.

## 1. Introduction

Intravascular lymphomas are rare malignancies in which the tumor cells are seen confined to the lumens of blood vessels. In this group of lymphomas, the most common is intravascular B-cell lymphomas (IVBCL). IVBCL has two clinical presentations: (1) the skin and central nervous system are affected in the classical variant, and (2) multiple organs are affected in the hemophagocytic syndrome-associated variant. Molecular studies have demonstrated mutations in B-cell receptor/NF-kappa B signaling, immune escape pathway, and PDL-1/2 in IVLBCL. In the rarer subtype, intravascular NK/T-cell lymphoma (IVNKTL), the tumor affects mainly the skin and central nervous system. Although the pathogenesis of IVNKTL is not currently known, Epstein–Barr virus (EBV) positivity is detected in the majority of reported cases, suggesting that EBV may play a key role in driving the disease process. Intravascular CD30+ lymphoma is exceedingly rare. In the histologic differential diagnosis are benign proliferations such as intralymphatic histiocytosis and intravascular atypical CD30+ T-cell proliferation. In this review, we present the clinical, histopathologic, and molecular findings of intravascular B-cell lymphoma, intravascular NK/T-cell lymphoma, intralymphatic histiocytosis, and benign atypical intravascular CD30+ T-cell proliferation. Since skin is the most accessible organ, obtaining biopsies for diagnosis would carry the least risk to the patients. Awareness of the cutaneous presentation of these rare intravascular hematolymphoid entities is of clinical importance.

## 2. Intravascular B-Cell Lymphoma

### 2.1. Clinical Presentation

#### 2.1.1. Epidemiology

Intravascular B-cell lymphoma (IVLBCL) is an aggressive extra-nodal non-Hodgkin lymphoma of the intravascular spaces [1,2,3,4,5]. It typically involves small and medium vessels [2,3], although it has been documented in all but major vessels [6,7]. The exact incidence is unknown [2]. It has been reported to be up to 1.5% of mature B-cell lymphoma cases in the Japanese Society of Hematology [8]. The United States Surveillance, Epidemiology, and End Results (SEER) 2000–2013 data study reported an age-adjusted incidence rate of 0.095 cases per million [3,4]. The median age at diagnosis is between 60 and 70 years old [1,2,3,6,9], with no sex predilection [1,2,3,4,6,9,10]. In the past, most patients were diagnosed with IVLBCL at autopsy [2,9]; however, recent data suggest approximately 80% of diagnoses are now made while patients are alive [6,11,12].

#### 2.1.2. Clinical Features

There are three distinct subtypes of IVLBCL: classical, hemophagocytic syndrome associated (HPS), and primary cutaneous type. Despite the fact that the presence of hemophagocytosis, rather than geographic location or race, distinguishes HPS from classical [5], the verbiage of “Western” to mean classical and “Asian” to mean HPS is perpetuated through the literature. This could lead to delays in diagnosis and treatment [13]; the classical and HPS variant terminology will be used in this paper. 

The clinical presentation of IVLBCL is variable, such that there is no “classic” clinical picture for IVLBCL [1]. Both the classical and HPS variants typically present with fever and a rapidly declining clinical course, with symptoms of organ dysfunction secondary to vascular occlusion [2,3,6]. Lymphadenopathy is absent in most cases [2]. Although formally classified as extra-nodal, 4–17% of cases may have nodal involvement [5]. Lactic dehydrogenase (LDH) elevation is seen in many cases (87–97%) [1,2,10,11,14]. Thrombocytopenia and elevated serum IL2R are common [1,2,15]. Thyroid, hepatic, and renal involvement (15–20%) may aid in diagnosis or determining relapse/progression of disease [6,7]. 

The classical variant presents with non-specific B symptoms (55–82%) [1,2,11,16] and neurological symptoms of a rapidly progressive nature [2,6,10,11]. The central nervous system (CNS, up to 30–41%) and skin (up to 48%) [6,16] are the most commonly involved sites [1]. The presence of multiple cutaneous lesions was correlated with worse outcomes in one study [10]. An additional 25% of patients presented with CNS involvement at subsequent follow-up post-diagnosis and/or post-therapy [2]. In the HPS variant, patients typically present with liver and/or spleen organomegaly [5]. Thrombocytopenia (76–84%), hypoalbuminemia (84%) [2], and bone marrow involvement are common (up to 75%) [6,17]. The primary cutaneous subtype tends to present with erythematous plaques/patches, on the trunk and/or lower extremities, in younger females and has a better prognosis than the classical and HPS variants [1,2,6,7,10,16].

Although clinical presentation is variable, many patients are incidentally diagnosed with IVLBCL in the setting of another neoplasm [1,9]. Reports of IVLBCL in the setting of mycosis fungoides [18], paraneoplastic syndrome [19], prostatic nodular hyperplasia [20], adrenal mass [21], and even acalculous cholecystitis [22] highlight the importance of an awareness of IVLBCL.

#### 2.1.3. Diagnostics

Despite being a malignancy of vascular spaces, 90–95% of cases do not have circulating IVLBCL cells in peripheral blood, making identification diagnostically challenging [1,6,7].

Position emission tomography/computed tomography (PET/CT) scans have helped localize disease for optimal biopsy sites [1,23,24]. Zhao et al. [24] examined 42 patients (2013–2022) who had undergone 18F-FDG (fludeoxyglucose) PET/CT prior to IVLBCL diagnosis. In total, 73.8% of patients had FDG-avid lesions detected. They compared FDG-avid lesions to those without and found similar clinical and histological features; however, there was a statistically significant difference in Ki-67 expression: the FDG-avid lesions had a lower average Ki-67 at 80%, compared to 90% in the non-avid group [24]. This highlights the utility of the FDG-PET scan; however, a negative scan does not confer a better prognosis for patients nor represent negative results. 

A definitive, minimally invasive, biopsy location was investigated. As a substantive portion of patients had cutaneous manifestations (up to 48%) [6,16], skin biopsies were explored with mixed evidence. Cho et al. [25] reported limited utility in random skin biopsies (0% positivity) in their HPS cohort. Another cohort study [15] examined the utility of random incisional biopsies and reported a 77.8% sensitivity and a 98.7% specificity [15]. Hemangioma biopsy has been shown to increase the likelihood of capturing involved vessels [4,26]. Rozenbaum et al. [4] provided biopsy recommendations: at least 4 mm of depth, sampling cutaneous abnormalities, and 3–6 samples of normal-appearing skin to provide a high-specificity diagnosis [4]. An additional study [27] concluded no utility in skin biopsy with normal LDH and sIL-2R less than 2000 U/mL. In a recent case report [28], a patient with increased LDH and CNS symptoms (negative imaging) was only diagnosed with IVLBCL after a random skin biopsy; this cautionary example highlights random skin biopsy utility. The conclusion from the literature is that adequate sampling (quantity and depth), paired with targeted biopsy (hemangiomas when available), has utility in the diagnosis of IVLBCL; however, a negative biopsy does not exclude this entity, particularly in the setting of the HPS variant [1]. 

As peripheral blood often lacks sufficient neoplastic cells [1,6,7], liquid biopsy for cell-free DNA (cfDNA) has been utilized on a small cohort (9 patients) to identify tumor-specific mutations [29]. The sensitivity to detect tumor-derived DNA (tdDNA)-identified mutations in cfDNA was reported as follows: 85% in diffuse large B-cell lymphoma (DLBCL) versus 100% in IVLBCL [29]. Shimada et al. [30] reported a statistically significant higher concentration of cfDNA in IVLBCL compared to other activated DLBCL samples. This methodology, if further validated, could allow the serum to be used as a test for IVLBCL. 

Lugano modification of the Ann Arbor staging system has been proposed for IVLBCL [1]. It has been suggested that all IVLBCL be treated as high-stage, regardless of imaging [7]. A recent review by Breakell et al. [2] even suggests that a specific prognostic index, inclusive of skin biopsy results, may be beneficial. 

#### 2.1.4. Treatment and Outcomes

The cutaneous variant has the best prognosis while the HPS variant carries the worst. A 46% 2-year overall survival in classical type IVLBCL (in the setting of anthracycline-based chemotherapy) and 30% 2-year overall survival (classical and HPS) were reported [5], comparable to the reported 5-year overall survival of 46.1% [3]. Shimada et al. [8] phase 2 trial of R-CHOP (rituximab, cyclophosphamide, doxorubicin, vincristine, and prednisone) plus methotrexate and intrathecal chemotherapy in patients with untreated non-CNS involved IVLBCL reported that 37 patients had a 2-year overall survival of 92% and progression-free survival of 76%. Compared to rituximab chemotherapy regimens without intrathecal therapy, secondary CNS involvement in this trial was 3% compared to 22% [8]. 

### 2.2. Histopathology

**Figure 1:**
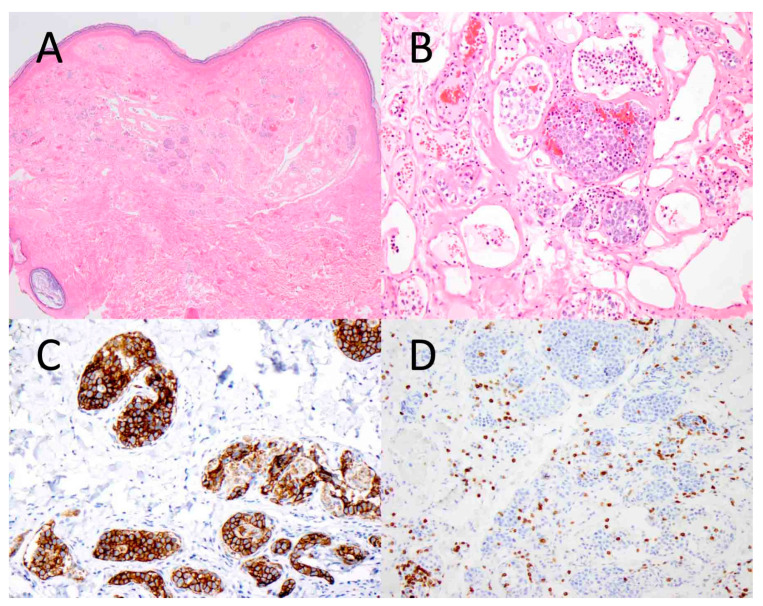
Intravascular B-cell lymphoma involving the skin. Atypical lymphoid cells are seen within the vascular lumens of a cutaneous hemangioma ((**A**), ×40). At higher magnification, the tumor cells are large and hyperchromatic, and they fill the vascular spaces ((**B**), ×200). The tumor cells express CD20, a B-cell marker ((**C**), ×200), while they are negative for CD3, a T-cell marker ((**D**), ×200).

Histologically, all variants contain large atypical lymphoid cells with prominent nucleoli (sometimes multiple), smooth contours, open chromatin, and abundant mitoses within the intravascular space (Figure 1) [6,20]. HPS tumors additionally contain a histiocytic background, containing cytoplasmic mononuclear and/or red blood cells [1,6]. 

### 2.3. Immunophenotype 

In the 2007 International Consensus meeting on IVLBCL, the importance of immunophenotyping was stressed to distinguish IVLBCL from vascular spread from another tumor or T-cell lymphoma [7]. In the recent World Health Organization (WHO) classification, IVLBCL was grouped in the large B-cell lymphomas category [31]. The tumor cells of IVLBCL express CD20 (99–100%), Pax5 (100%), CD79a (100%), and other B-cell markers such as CD19, BOB.1, and OCT2 [2,14,16,32]. BCL2 (80–100%) [11], CD10 (13%) [17], and MUM1 (75–100%) positivity [14,16,33], with varied positivity for BCL6 [16,33], were reported. Positivity for MYC (68–71.4%) [16,33] and IgM (91%) [16] was reported. CD3 is importantly negative and distinguishes IVLBCL from T-cell lymphoma [2].

One potential pitfall is the expression of pan T-cell marker CD5 (38–50%) [11,16,33,34,35]. The largest review of 342 cases showed 45.1% CD5 positivity [14]. An older article reported a 38% CD5 positivity, which was associated with bone marrow, splenomegaly, and peripheral blood involvement; 93% had hemophagocytosis, which suggests the possibility of a connection between CD5 positivity and the HPS variant [17]. More data are needed to elucidate a correlation between CD5 positivity and variant subtype.

**Figure 2:**
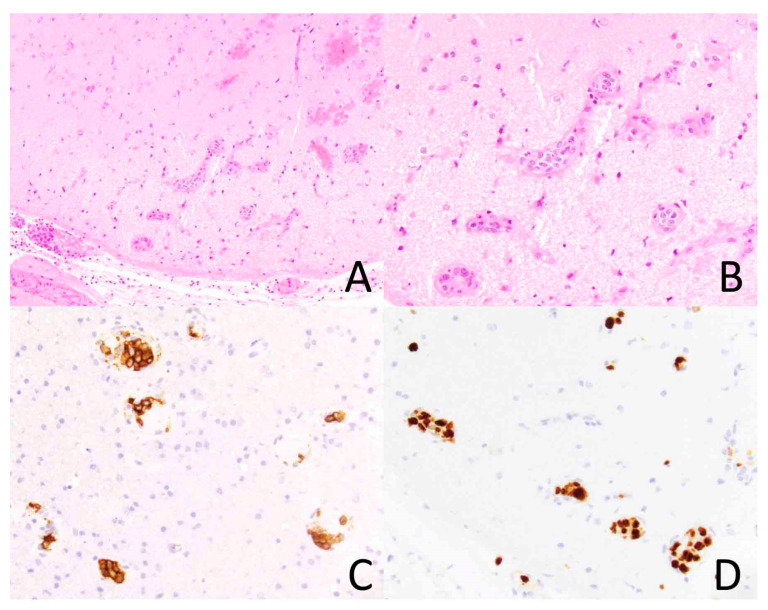
Intravascular lymphoma involving the brain. The vasculature of the brain parenchyma is dilated by atypical lymphoid cells ((**A**), ×200). At higher magnification, the tumor cells are large and hyperchromatic ((**B**), ×400). The tumor cells express CD20, a B-cell marker, ((**C**), ×400) and they exhibit a high Ki-67 proliferation index ((**D**), ×400).

Ki-67 expression level is typically high (Figure 2) [2,32]. One study [32] reported a Ki-67 proliferation index above 60% in all 17 cases; when stratified into “lower” (60–79% proliferation index) and “higher” (80–100% proliferation index), the one- and two-year survival rates were significantly different, 83.3% versus 60.0% one-year survival rate and 31.3% versus 0.0% two-year survival rate. 

Programmed death-ligand (PDL)-1 is another marker with variable positivity (36–44.4% of cases) [9,34,36], suggesting an immune evasion mechanism. More data are needed to understand the prevalence of PD-L1 in IVLBCL. 

A small subset of cases had Epstein–Barr virus (EBV)/ EBV-encoded RNA (EBER) positivity; one study reported 7.7% positivity [14], but other studies reported 100% negativity [17]. Further data are necessary to determine the significance of EBV/EBER positivity.

### 2.4. Molecular Findings

The molecular findings align with the immunophenotype. Bauer et al. [37] performed molecular testing via microarray on one case of (presumed) classical variant of IVLBCL, and the molecular profiling was consistent with non-germinal center DLBCL; however, this study may not represent the heterogeneity within IVLBCL. 

#### 2.4.1. B-Cell Receptor/Nuclear Factor Kappa Beta Signaling

Targeted next-generation sequencing (NGS) [16], cfDNA [29], whole exome sequencing (WES) of patient-derived mice xenograft tumors, tumor DNA or bone marrow [30], fluorescence in situ hybridization (FISH), and NGS [9] were used to identify and confirm *MYD88* (44–57%) and *CD79B* (26–67%) mutations. Shimada et al. [30] reported a 43% concomitant *CD79B* and *MYD88* mutation rate. Both *MYD88* and *CD79B* are involved in the BCR/NF-kappa B signaling pathway, which may be an immune evasion mechanism by IVLBCL. 

Additional B-cell/nuclear factor(NF)-kappa B pathway mutations were reported by Shimada et al. [30]; WES included the following: *IRF4* (38%), *TNFAIP3* (24%), *NFKBIE* (14%), and *ITPKB* (14%). B-cell development mutations included *PRDM1* (43%) and *TOX* (33%). Chromatin-histone modification factor mutations included *SETD1B* (57%), *KMT2D* (24%), and *EP300* (14%). Patients with immune evasion-related genetic alterations and *MYD88* mutations had poorer outcomes [30]. These studies confirm that mutations in B-cell receptor/NF-kappa B signaling and immune escape pathway are present in IVLBCL, with one study reporting that the genetic profile was consistent across histologic variants, raising the potential therapeutic implications with NF-kappa B signaling-targeted therapy [9]. 

Patel et al. [36] reported MHC class I and or II expression loss in 27% of cases, representing an additional immune evasion mechanism. Shimada et al. [30] demonstrated *HLA-B* mutations by WES. Gonzalez-Farre et al. [9] reported *PIM1* mutation in 60% of cases (9/15). These mutations further suggest an immune evasion mechanism.

#### 2.4.2. PDL-1/2

Mutations in PDL-1 and/or PDL-2 have been identified in a subset of cases of IVLBCL. Shimada et al. [30] showed that 38% of the IVLBCL patients had PDL-1 and/or PDL-2 rearrangements. Patel et al. [36] reported that 45% (5/11) of IHC-positive cases had a chromosomal alteration in either PD-L1 or PD-L2 by FISH. They subcategorized the cohort by variant (11 HPS, 22 classical). All in all, 50% of HPS cases were PDL-positive compared to 33% of classical cases. PDL-2 expression was only seen in the HPS variant (2/10). Larger studies are needed to determine the prevalence of PDL-1/2 expression in IVLBCL, as this could have therapeutic implications. 

### 2.5. Pathogenesis

The pathogenesis of this entity has yet to be fully elucidated. Ponzoni et al. [20] showed that six cases of IVLBCL lacked the adhesion molecules CD29 and CD54. CD29 is beta-1 integrin, which is responsible for trafficking lymphocytes and trans-vascular migration. CD54 is ICAM-1, which is also responsible for trans-vascular migration. Additionally, low or absent levels of adhesion molecules and matrix metalloproteinases in IVLBCL were reported [22]. The absence of these molecules suggests that an inability to migrate across the vascular space is one mechanism in the pathogenesis of IVLBCL. Mutations in immune evasion pathways and *PDL1*/*PDL2* suggest that immune evasion may also be involved [9,30]. Shimada et al. [38] took samples from IVLBCL xenograft mice tumor cells and showed that they had preferential growth in environments rich in endothelial cells, which suggests that the vascular endothelial environment is in part responsible for the growth pattern of IVLBCL. These authors crossed tumor-derived cells to different organs in subsequent mice, and their results suggest an organ-specific environmental preferential growth for IVLBCL [38]. Xenografted mice with IVLBCL (confirmed through immunoprofiling) underwent gene set enrichment analysis of tumor cells indicating suppression of myosin pathway genes responsible for cell migration, which may be involved in the pathogenesis, although further studies on larger cohorts are necessary [38].

IVLBCL is found in the vessels of benign neoplasms in 15% of patients [6], or in conjunction with other lymphomas or solid tumors [10,18,39]. This characteristic finding of cells within another neoplasm suggests that benign neoplasia produces molecules that promote IVLBCL growth. 

### 2.6. Summary

The pertinent clinicopathologic features of IVLBC are outlined in Table 1. Molecular studies confirm mutations in B-cell receptor/NF-kappa B signaling, immune escape pathways, and PDL-1/2 are present in IVLBCL. With the incidence of IVLBCL increasing [3], more epidemiologic, clinical, and molecular data may assist clinicians and researchers to better elucidate the clinical course, pathogenesis, and treatment regimens for these patients. 

## 3. Intravascular T/NT Lymphoma

### 3.1. Clinical Presentation

Intravascular natural killer/T-cell lymphoma (IVNKTL) is a highly aggressive form of non-Hodgkin lymphoma that primarily impacts the CNS and the skin [40]. It typically affects small to medium-sized vessels and does not form a mass lesion [40]. IVNKTL is an extremely rare disease, with a recent literature review indicating that just 27 cases with sufficient immunohistochemical and molecular data have been reported since the disease was first described by Santucci et al. in 2003 [41,42]. Given its rarity, optimal disease classification remains unclear; IVNKTL was formerly considered to be a variant of extranodal NK/T-cell lymphoma [43,44]; however, the most recent edition of the WHO classification of lymphoid neoplasms categorizes IVNKTL as a subtype of aggressive NK-cell leukemia instead [45]. IVNKTL has predominantly been reported in adult patients without a clear age or sex predilection [40,42]. 

The clinical features of IVNKTL are highly variable, contributing to challenges in making the diagnosis. The vast majority of patients present with skin findings, though the rash characteristics follow no consistent pattern [42]; erythema [46] and erythematous plaques or nodules [47,48,49], sometimes with overlying telangiectasias [50], are frequently described in the literature. In addition to the cutaneous manifestations, other organs and organ systems including the CNS, bone marrow, lungs, and kidneys may be affected, with CNS manifestations being the most common [46,47]. Due to the potential for many different organ systems to be involved, presentation is highly variable; symptoms may include fever and other B-symptoms, malaise, jaundice, neurologic symptoms, arthralgias and other musculoskeletal symptoms, or rash [40,46,47,51]. To further complicate matters, some patients present only with cutaneous manifestations with no symptomatic evidence of systemic disease [40,42,48,52]. Hematologic findings including anemia, thrombocytopenia, and leukopenia are sometimes observed upon workup due to involvement of the bone marrow [33,48].

#### 3.1.1. Diagnosis

Skin biopsy is the most effective means of diagnosis [42]. In cases without evidence of skin involvement, patients may alternatively be diagnosed via bone marrow [33] or brain [42] biopsy. 

A recent article composed by members of the American Registry of Pathology recommends the use of both flow cytometry and in situ hybridization in the workup of NK/T-cell lymphomas involving the blood and bone marrow. The flow cytometry antibody panel should include T-cell markers such as CD2, CD3, CD4, CD5, CD7, and CD8, as well as antibodies to TCR alpha/beta and gamma/delta [53]. The inclusion of CD16, CD56, CD57, and CD94 is additionally recommended, given that NK disease is on the differential [53]. EBER in situ hybridization should be utilized for the detection of EBV [53]. 

#### 3.1.2. Treatment and Prognosis

IVNKTL is a highly aggressive disease associated with an extremely poor prognosis; average survival is weeks to months [54,55]. There is no standard treatment protocol at this time, which is likely due to the rarity of the disease. In general, traditional cancer therapies including chemotherapy and radiation are ineffective [56]. CHOP therapy in particular has been noted to be unsuccessful in the treatment of IVNKTL due to inherent disease chemoresistance [40,48,57,58]. Some cases have been observed to respond more favorably to treatment with proteasome inhibitors or salvage chemotherapy (e.g., DHAP: dexamethasone, cytarabine, cisplatin) [40]. The available literature suggests that anthracycline-based chemotherapy coupled with stem cell transplantation may offer the most effective treatment [48], though only one case of long-term survival (8 years as of this writing) following stem cell transplant has been reported [59]. A 2019 genetic study of IVNKTL found strong expression of PD-L1, suggesting a potential role for immune checkpoint inhibitors in the treatment of this disease [40,60].

Prognosis has been suggested to be more favorable in patients presenting solely with skin involvement in comparison to those with multisystem involvement, though even those patients with primarily cutaneous disease have a very unfavorable outlook [40,47,48,56].

### 3.2. Histopathology

Histology typically demonstrates a proliferation of atypical lymphoid cells containing irregular, elongated nuclei with single or multiple small nucleoli and pale or eosinophilic cytoplasm within the vascular lumen, sometimes with an inflammatory infiltrate surrounding the involved vessels [56,57,60,61]. Small to medium-sized vessels are most often impacted by the disease process, and the malignant cells do not form a mass [40]. Mitotic figures may be easily visualized [52,60,62].

### 3.3. Immunophenotype

The neoplastic cells express CD2, CD3ε, CD56, and cytotoxic molecules including TIA-1, perforin, and granzyme B [46,48,62,63]. The tumor cells typically do not express surface CD4, CD5, CD8, betaF1, or TRC delta [46,48,62,63]. Occasionally, expression of CD7 or CD30 is present [46,48,62,63]. Diffuse positivity for EBER by in situ hybridization is seen [46,48,62,63]. The majority of cases are TCR gene rearrangement negative [56].

### 3.4. Differential Diagnosis

The differential diagnosis of IVNKTL is broad and includes the following: extranodal NK/T-cell lymphoma, nasal type; aggressive NK-cell leukemia; EBV-positive nodal T/NK-cell lymphoma; intralymphatic CD30+ large T-cell lymphoma; benign atypical intralymphatic CD30+ T-cell proliferation; and intravascular large B-cell lymphoma. Each of these entities is discussed briefly here. 

The main disease process from which IVNKTL must be differentiated is extranodal NK/T-cell lymphoma, nasal type (ENKL). ENKL most commonly affects the nasopharynx and upper aerodigestive tract, though the skin may additionally be involved [64]. It is most frequently observed in East Asian and Latin American countries and only rarely diagnosed in the United States, where the incidence is greatest in Hispanic white Americans and Asian/Pacific Islanders [65]. ENKL is primarily a disease of middle-aged adults with an observed male-to-female predominance of approximately 2:1 [65,66]. Patients initially present with localized symptoms including nasal congestion and epistaxis before experiencing systemic symptoms such as weight loss and fever [64,67,68,69]. Though tumor cells may be present intravascularly [70], raising initial concern for IVNKTL, they are not confined to the vascular system and have the potential to infiltrate surrounding tissues via angioinvasion [48]. Tumor cells typically have an NK-cell phenotype, expressing CD2, CD3ε, and CD56, with no expression of surface CD3, CD4, or CD5 [64]. Expression of CD25, CD30, CD38, FAS, and FASL is variable [64].

Aggressive NK-cell leukemia presents with a similar clinical and immunophenotypic picture to IVNKTL, though skin involvement is less common, and is also associated with EBV positivity [40,47]. It usually presents in young to middle-aged adults [40]. In this disease, malignant cells are not confined to the vasculature and are instead widely distributed in peripheral blood, bone marrow, liver, and spleen [47,52]. The immunophenotype is the same as ENKL, except with more frequent CD16 expression [40].

EBV-positive nodal T/NK lymphoma is a systemic lymphoma that primarily affects older adult patients [40,71]. The disease presents with lymphadenopathy and sometimes with B symptoms and evidence of extranodal involvement, such as hepatosplenomegaly [40,71]. The majority of cases (80%) are of T-cell lineage, with the remainder being of NK-cell lineage; tumor cells are therefore typically positive for T-cell markers and cytotoxic molecules [40,71]. The vast majority of cases are positive for EBV [40,71]. In contrast to IVNKTL, this disease shows infrequent CD56 expression and an absence of angioinvasion [40,71].

Intralymphatic CD30+ large T-cell lymphoma presents as plaques or tumors with surrounding satellite lesions and, notably, an absence of telangiectasia [50]. Tumor cells express CD3, CD4, and CD30; cytotoxic molecules and EBER are negative [50]. Malignant cells are confined to lymphatic vessels, as opposed to blood vessels, as demonstrated by positive D2-40 staining of involved vessels [72]. In comparison to IVNKTL, which stains negative, involved vessels stain positive for podoplanin [50]. 

IVNKTL should also be differentiated from benign atypical intralymphatic CD30+ T-cell proliferation and intravascular B-cell lymphoma, which are discussed in detail elsewhere in this review. 

### 3.5. Molecular Findings

A 2019 study by Fujikura et al. analyzed the genetic features of IVNKTL using whole exome sequencing and identified frequent mutations in genes coding epigenetic regulators, including four histone genes (*HIST1H2BE*, *H3F3A*, *HIST1H2BN*, and *HIST1H2AM*) and two DNA methylation genes (*TET2* and *DNMT1*), suggesting that failures in epigenetic regulation may contribute to disease development [60]. Strong expression of PD-L1 was also identified [60]. A follow-up study published in 2020 investigating alternative splicing patterns in IVNKTL identified mutations in 15 splicing regulator genes (e.g., *SF3B5*, *SRSF12*, and *TNPO3*) in addition to previously identified alterations in known tumor suppressors and oncogenes (e.g., *HRAS*, *MDM2*, and *VEGFA*), indicating that aberrant splicing may additionally contribute to the disease process [73]. One case of IVNKTL analyzed by karyotyping by the same group was found to have a normal karyotype [74].

### 3.6. Pathogenesis

The pathogenesis of IVNKTL is not understood. However, EBV positivity has been noted in nearly all reported cases, suggesting that EBV may play a key role in driving the disease process [48,63]. As discussed in the previous section, genetic studies of IVNKTL have identified mutations in the epigenetic regulator and splicing regulator genes, suggesting that a complex interaction involving failures of epigenetic regulation and aberrant splicing mechanisms may drive disease development. The precise role of EBV infection in this process warrants further investigation. 

The tendency for IVNKTL to grow exclusively within the vasculature has been hypothesized to be driven by mutations in adhesion molecules such as I-CAM and ITGB1; however, additional investigation is needed to confirm the mechanism of the growth pattern of this disease [49,60].

### 3.7. Summary

The pertinent clinicopathologic features of IVNKTL are outlined in Table 2.

## 4. Intralymphatic Histiocytosis

### 4.1. Clinical Presentation 

Intralymphatic histiocytosis (IH) was first described as “intravascular histiocytosis” by O’Grady in 1994 in a 77-year-old female with a rash on the lower extremity [75]. Since then, there have been numerous studies published with similar histopathologic findings but with further clarification on the intralymphatic nature of the involved vessels. Clinical presentations are diverse. Bakr and colleagues [76] posited that IH can be secondary to systemic disease or occur without inciting factors. Indeed, two patients in their study who had no evident systemic process occurring at the time of skin biopsy were eventually diagnosed with IH. Regarding associations with systemic disease, rheumatoid arthritis is the most common disease association [76,77,78,79,80,81,82,83,84,85], but IH has also been reported in osteoarthritis [86,87], Merkel cell carcinoma [81], mastectomy sites [81,88], joint replacements and metal implants [81,84,88,89,90], breast implants [91], melanoma [81], Klippel–Trenaunay syndrome [81], Crohn’s disease [76], cellulitis [92], and genital swelling and necrosis [84,93]. 

Patients range from 17 to 87 years (mean age, 66 years) at diagnosis [76,91]. With regards to skin findings, presentations can be diverse and be present for years before biopsy [83,94]. Especially in the setting of rheumatoid arthritis, common findings include irregular, livedo-like patches of erythema proximal to swollen joints in patients with rheumatoid arthritis [79,80,81,83,94,95]; however, the trunk can also be involved with similar lesions [82]. Swelling and indurated plaques have also been reported, including on the limbs, trunk, eyelids, lips, and genital areas [76,81,83,94,95]. Outside of rheumatoid arthritis, skin findings can present differently, as in the case of a patient who developed widespread red to violaceous patches after a breast implant [91] or a patient with a smoking history and Legionnaires disease who developed facial swelling eventually diagnosed as IH [95].

### 4.2. Treatment

Although a benign process, intralymphatic histiocytosis is indolent and typically refractory to treatment. Due to the difficulties of diagnosis, the rarity of the condition, and the diverse clinical contexts in which IH arises, there is no standard treatment. Patients with this condition have been treated with immunosuppressants such as corticosteroids [77,81] and electron beam radiation [75] but with recurrence. It seems to be the case that treating co-existing rheumatoid arthritis can improve the IH lesions, and this has been done with methotrexate [81], NSAIDS plus steroid injections into the joint [79], and infliximab [94]. In one case, amoxicillin plus acetylsalicylic acid was used with good effect [83]. In the case of swollen joints or lymphedema, compression wrappings have been found to be helpful [77,85]. Apart from compression wrappings, skin-directed therapies have been largely unhelpful [77].

### 4.3. Histopathology

**Figure 3:**
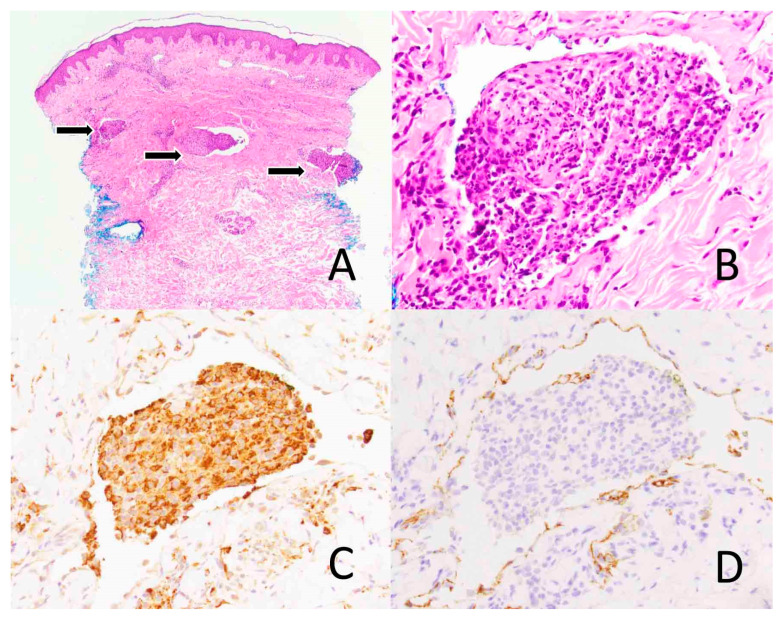
Intravascular histiocytosis. Skin biopsy shows the dermal vascular spaces expanded by lymphoid cells (arrows) ((**A**), ×40). The infiltrate is comprised mainly of histiocytes ((**B**), ×400) that express CD68, a macrophage marker, ((**C**), ×400) and lymphocytes. D2-40, a lymphatic marker, highlights the dermal vascular channels ((**D**), ×400).

The pathologic finding unifying these diverse clinical presentations is dilated vessels with intraluminal collections of histiocytes within the reticular dermis (Figure 3). Granular cytoplasm characteristics of histiocytes have also been reported [75,77,81]. Rieger found that the medium and large intraluminal cells formed “glomeruli”-like structures; lysosomal and phagocyte-like structures on electron microscopy confirmed the presence of intraluminal histiocytes [77]. Consistent with their histiocytic identity, intraluminal cells are mononuclear, with ovoid, indented nuclei [77,81]. Both Rieger and Pruim noted the irregularity of the vessel walls, which the latter thought was more in keeping with lymphatic vessels rather than blood vessels [77,78]. Bakr noted dermal edema, fibrosis, and granuloma-like collections of histiocytes [76]. In some cases, IH showed granulomatous inflammation in the dermal interstitium surrounding the dilated vessels [82,94]. Histiocytes are not the only cell type that has been noted within these collections. To a lesser extent, plasma cells, lymphocytes, and neutrophils have been also noted [76,80,81,82]. Perivascular infiltrates are also common and can include histiocytes, plasma cells, lymphocytes, neutrophils, and even eosinophils [76,78,79,81,82,83].

### 4.4. Immunophenotype

O’Grady’s earliest reported case included cells strongly staining for MAC 387 and KP1 (CD68), which are classic macrophage markers. While there was weak staining for CD45, a potential marker for lymphomas or leukemias, other B- and T-cell markers typical of lymphomas were negative [75]. Since that publication, CD68 has been the dominant marker for confirming the presence of histiocytes, although older macrophage markers, namely, MAC 387 and HAM 56, have been found to be positive [77,78,81]. HLA-DR, a marker for inflammatory activation, has also been tested and reported as positive [76,77]. A limited number of cells have been positive for common B and T lymphocyte markers (CD45RO, CD3, CD20), confirming that lymphocytic involvement is minor if not absent [78]. Endothelial markers (CD31, CD34, factor VIII-related antigen) for the cells within the lumen have also been largely negative [77,79]. 

There has been debate about whether these cases represented intravascular or intralymphatic collections of histiocytes, as in the past, only common vascular and lymphatic endothelial markers were available (CD31, CD34, factor VIII) [75,77,83]. While it has been already mentioned here that the irregular vessel walls clued in some to the endothelial cells’ lymphatic origin, the advent of the D2-40 antibody, a highly-specific marker for lymphatic endothelial cells, made distinguishing between the two possible and confirmed lymphatic origin [76,80,81,94,96,97].

### 4.5. Differential Diagnosis

The differential diagnosis of IH includes the following: intravascular lymphoma, leukemia, reactive angioendotheliomatosis, intravascular histiocytosis, malignant histiocytosis, and intravascular spread of carcinoma or melanoma. IH can be distinguished from lymphoma and leukemic infiltrates by both immunohistochemical findings and clinical presentation. In particular, IH would show dominant histiocytes within the intraluminal collections. The restriction of endothelial markers by immunohistochemistry to the vascular walls excludes reactive angioendotheliomatosis, a benign intraluminal proliferation of endothelial cells. However, this entity in the differential is controversial for reasons that will be explained further below. While IH is benign and follows an indolent clinical course, leukemia and lymphoma have a more aggressive clinical behavior with likely systemic involvement.

### 4.6. Molecular Findings

To our knowledge, no molecular studies have been conducted on samples of patients with IH.

### 4.7. Pathogenesis

There are several theories about the pathogenesis of the condition. Rather than being a distinct diagnosis, some have questioned if the intravascular histiocytic collection seen on skin biopsies is a precursor to reactive angioendotheliomatosis, a benign proliferation of endothelial cells associated with a myriad of systemic disorders [77,98]. Rieger theorized that in the event of a systemic disease, vascular occlusion could lead to endothelial activation and attraction of monocytes like histiocytes, forming the “glomeruloid” structures seen on biopsy [77]. Neither Rieger nor others have been able to provide sequential evidence of this progression from intraluminal histiocytic collections to endothelial proliferation. In response to the confusion about the differences between reactive angioendotheliomatosis, intravascular histiocytosis, and intralymphatic histiocytosis, Mazloom et al. [99] analyzed reported cases of all three and concluded the following: that the reactive angioendotheliomatosis and intravascular histiocytosis share clinical presentations and histopathologic findings, and that intralymphatic histiocytosis is a distinct clinical and histologic entity. Altogether, these findings caution against the usage of intravascular and intralymphatic histiocytosis as interchangeable terms due to consequent differences in treatment and outcomes.

Requena and Pruim have drawn attention to the importance of chronic inflammation contributing to blockage in lymphatic drainage [78,81]. Supporting this notion is the therapeutic response to infliximab in the treatment of cutaneous lesions of intravascular histiocytosis [94]. This theory is especially relevant in patients with histories of arthritis and surgeries that often alter lymphatic flow, as the lesions typically develop in areas of swollen joints or chronic lymphostasis [78,79,81,94]. Some have surmised that this chronic lymphostasis contributes to the pro-inflammatory state necessary to trigger IH [94,100]. Asagoe surmised that there might be two benign entities characterized by intraluminal collections of histiocytes: one in the lymphatic vessels—a condition typical in rheumatoid arthritis-and one in blood vessels [101]. The latter is proposed to be in alignment with what was theorized by Rieger as the first step in reactive angioendotheliomatosis and is consistent with the bacterial infection and consequent inflammation seen in a patient with tonsillitis [77,101].

### 4.8. Summary

The pertinent clinicopathologic features of intralymphatic histiocytosis are outlined in Table 3.

## 5. Benign Atypical Intravascular CD30+ T-Cell Proliferation

There is a spectrum of diseases associated with CD30-positive cells, spanning from benign reactive processes to malignant, aggressive clinical presentations. This review focuses on presentations falling in the middle of this spectrum: benign, atypical intravascular (including intra-lymphatic) CD30-positive proliferations. Under physiological conditions, CD30 is usually expressed on follicular B cells and is thought to be a marker of activated T cells, including T2 helper T cells and a fraction of activated CD45RO+ memory T cells [102,103]. Atypical CD30-positive, lymphoid cells can be found in a broad scope of contexts, including inflammatory, infectious, environmental, or drug eruptive processes [104,105]. 

When CD30 positivity is paired with atypical lymphoid cells within the vasculature, clinicians and pathologists are rightly concerned about the possibility of malignancy. However, there has been a growing consensus about the often-benign course of this entity.

### 5.1. Clinical Presentation

A constellation of reports has added to a growing consensus on the possibility of a benign clinical course with atypical CD30-positive T cells. In one early study on this entity, Baum reported a case of a 17-year-old male presenting with a 2-month history of ulcerative lesion initially thought to be pyogenic granuloma that developed after trauma but whose biopsy revealed the atypical lymphocyte infiltrate that is the subject of this current review [106]. 

More recent studies have highlighted similarly diverse clinical presentations. A case report from 2019 featured a patient with primary diffuse large B-cell lymphoma who developed a genital ulcer after starting chemotherapy. While initially thought to be intravascular lymphoma, the lesion subsequently regressed and was determined to be an atypical CD30+ T cell proliferation, without any new eruptions [107]. Ulcerative or pyogenic granuloma-like skin findings are common clinical descriptors and have occurred in the genital area in response to trauma [108], on the trunk without provocation [108], in the context of lichen sclerosus [109], in a cutaneous hemangioma [110], in the oral cavity [111], and in the excised tissue of a patient with hidradenitis suppurativa [112]. Intravascular atypical CD30-positive T cells have also been found in lesions following drug initiation. Weingertner reported a case of a 77-year-old man who developed a prurigo-like, maculopapular exanthem of the trunk and was subsequently diagnosed with drug reaction with eosinophilia and systemic symptoms (DRESS) but had a skin biopsy with atypical intralymphatic CD30-positive lymphocytes. Two other cases occurred in the context of immune checkpoint inhibitor (ICI) therapy, one with a morbilliform eruption, and the other with a more eczematous presentation [113,114].

### 5.2. Treatment

Due to the paucity of recorded cases and diversity of clinical presentations, there is no standard treatment for this condition. Most case reports of this condition indicate that the lesions resolved on their own without recurrence [106,107,108,109,111,115]. In the cases of cutaneous toxicities with immune checkpoint inhibitor (ICI) therapy, the lesions resolved with oral and topical steroids [113,114].

### 5.3. Histopathology

The defining histopathologic feature of this condition is CD30-positive T cells within the vasculature, either blood vessels or the lymphatic system. Aside from these features, a variety of findings have been reported. Other histopathologic findings include dermal edema [106,114,115], epidermal hyperplasia [108,114], epidermal spongiosis [115], and hyperkeratosis [115] (the latter two in the context of DRESS). 

In addition to CD30 positivity, T cells in reported samples are predominately CD4 positive [106,108,109,110,111,114,115]; however, one exception is a case of ICI-induced eruption, which stained positive for CD8 [113]. Ardighieri et al. [110] found T-expressing markers characteristic of effector or memory-like T regulatory cells (positive for CD45RO and FOXp3), as well as CCR7, indicating a lymph node homing phenotype. Pleomorphic and small nucleoli have been reported [107], as well as mitotic figures, apoptotic bodies, and high proliferation index marker Ki-67 [108,110,111,113,115]. Some studies have sought to identify the vessels in which the atypical lymphoid cells resided and found them to be lymphatic, with positive D2-40 staining [109,110,111,112,115]. Riveiro-Falkenbach noted lobular vascular proliferation in two reported cases [108]. In the inflammatory infiltrate in the perivascular, dermal space, various cells have been found, including neutrophils, plasma cells, lymphocytes, eosinophils, and histiocytes [108,109,113].

In light of the diagnostic difficulties, Kempf proposed diagnostic criteria for atypical intravascular CD30-positive T-cells [109]. The criteria emphasize an association with trauma, ulceration, or inflammatory processes, coupled with intralymphatic accumulation of medium- to large-sized activated lymphocytes. Essential features comprise the expression of T-cell markers and CD30 without B-cell marker expression. Additional criteria encompass the absence of loss in T-cell markers (except for CD7), lack of Epstein–Barr virus (EBV) involvement, and no clonal rearrangement of T-cell receptor (TRC) genes. Staging examinations rule out indications for cutaneous or systemic lymphoma, and an indolent course with complete resolution post-ulceration or inflammation characterizes the condition, with no subsequent lymphoma development during follow-up. While these criteria may not completely resemble what is seen in pathology, they provide a comprehensive framework for diagnosing an atypical intravascular CD30-positive T-cell proliferation and ruling out malignancy.

### 5.4. Differential Diagnosis

The differential diagnosis for atypical intravascular CD30+ T-cell proliferation involves considering several entities with overlapping features. Distinguishing features from intravascular anaplastic large cell lymphoma (ALCL) and lymphomatoid papulosis (LyP) can be challenging, especially since the line between CD30+ T-cell lymphoproliferative disease and benign CD30+ T-cell disorders is not well-defined. Samols attempted to use *DUSP22*::*IRF4* fluorescence in situ hybridization to accurately categorize intralymphatic lymphocyte collections as anaplastic lymphoma, but negative or omitted results underscore the difficulty in differentiation [72]. Additionally, intravascular lymphoma, particularly large T-cell subtypes with CD30 positivity, follows an aggressive course, making careful assessment and staging crucial [116,117]. Leukemia cutis, characterized by cutaneous infiltration of leukemic cells, should also be considered in the context of CD30+ T-cell proliferation. Monitoring for signs of cutaneous or systemic lymphoma development during follow-up is essential, as an indolent course with complete resolution after regression of ulceration or inflammatory processes is indicative of atypical intravascular CD30+ T-cell proliferation. The integration of clinical, histopathological, and molecular findings is critical for accurate diagnosis, necessitating a comprehensive approach for effective patient management and monitoring over time.

### 5.5. Molecular Findings

Utilization of molecular diagnostics in reported cases has largely been restricted to clonality studies. In the diverse inflammatory, infectious, and environmental skin biopsy samples studied by Cepeda, CD30+ atypical T lymphocytes often had polyclonal TCR gene rearrangements. Other studies have similarly found oligoclonal or polyclonal TCR gene rearrangements [106,107,108,109]; however, some cases, in particular, the ones in which a drug was implicated, have not [114,115].

### 5.6. Pathogenesis

Much is unknown about the etiology of this condition. Kempf posited that trauma or inflammatory states serve as the trigger for a cascade of immunomodulatory signaling that results in the CD30+ T cell intralymphatic collections and perivascular infiltrates seen on biopsy [109]. While Kempf referred specifically to the cases with ulcerative lesions, this theory has been cited by authors reporting on CD30+ T-cell, intralymphatic infiltrates to morbilliform drug rashes [113,114,115]. 

### 5.7. Summary

The pertinent clinicopathologic features of benign atypical intravascular CD30+ T-cell proliferation are outlined in Table 4.

## Figures and Tables

**Table 1:** Summary of intravascular B-cell lymphoma.

Clinical features
There are three clinical subtypes of IVLBCL: classical type, hemophagocytic syndrome associated type, and primary cutaneous type. Biopsy hemangiomas has been shown to increase the likelihood of capturing the involved vessels, but adequate sampling including subcutaneous adipose tissue increases the chance of capturing IVLBCL even in normal-appearing skin.
Histopathology
Immunophenotypic findings show expression of B-cell markers including CD20, CD79a, and Pax5. Negative for T-cell markers. CD5 expression can be seen in 38–50% of cases.

**Table 2:** Summary of intravascular natural killer/T-cell lymphoma.

Clinical features
Highly variable; typically affects the skin and central nervous system, with erythematous rash and neurological symptoms common. Prognosis is very poor; weeks to months.
Histopathology
The neoplastic cells express CD2, CD3ε, CD56, and cytotoxic molecules including TIA-1, perforin, and granzyme B. They typically do not express surface CD4, CD5, CD8, betaF1, and TRC delta. EBV/EBER is invariably positive.

**Table 3:** Summary of intralymphatic histiocytosis.

Clinical features
Commonly associated with an underlying condition, most commonly rheumatoid arthritis, but also with other diverse triggers. Common skin findings include irregular, livedo-like patches of erythema proximal to swollen joints and issue, as well as indurated plaques.
Histopathology
Dilated lymphatic vessels with intraluminal collections of histiocytes. Immunophenotypic findings include CD68 positive cells (characteristics of histiocytes) within D2-40 positive endothelial vasculature (indicating lymphatic origin).

**Table 4:** Summary of benign atypical intravascular CD30+ T-cell proliferation.

Clinical features
Ulcerated lesions form in both patients with cancer and otherwise healthy individuals. Morbilliform eruptions can also form in the context of drug initiation.
Histopathology
Immunophenotypic includes CD30-positive T cells, with most displaying a CD4 predominant pattern, within the D2-D40 lymphatic vessels.

## Data Availability

Not applicable.
